# Sleep Telemedicine: A Survey Study of Patient Preferences

**DOI:** 10.5402/2012/135329

**Published:** 2012-07-09

**Authors:** Jessica M. Kelly, Lee H. Schwamm, Matt T. Bianchi

**Affiliations:** Neurology Department, Massachusetts General Hospital, Wang 720, 55 Fruit Street, Boston, MA 02114, USA

## Abstract

Telemedicine is an increasingly recognized option for cost-effective management of chronic conditions. We surveyed Sleep Clinic patients about their experiences and preferences regarding different forms of telemedicine. Adult Sleep Clinic patients seen between 2009 and 2011 received a brief survey either by postal mail (*n* = 156) or, for those with an available email address, electronically (*n* = 282). The overall response rate was 28.1% (*n* = 123 responses), with email response rates being higher than postal mail responses. The most commonly reported barriers to in-person physician visits were parking cost (44%), time away from work/school (34%), and cost of gas (26%). Whereas 89% of respondents indicated using telephone and 55% of respondents indicated using email to communicate with providers, none reported experience with video telemedicine. Despite this lack of experience, over 60% reported feeling comfortable or willing to try it. Of those who were uncomfortable about video telemedicine, the two main reasons were that in-person visits feel more natural (48%) and that the doctor might need to perform an examination (24%). More than half of respondents reported willingness to pay a copay for a video visit. Video telemedicine represents a feasible option for chronic sleep disorders management.

## 1. Introduction

Telemedicine utilization is rising due to the increased availability and decreased cost of communication technology, in parallel with growing recognition of key areas of health care that may benefit from its use. Although phone communication continues to be a common form of patient-provider communication, internet-based video communication is being deployed in several settings as well. The portion of the population with internet access is rapidly growing—74% of English-speaking Americans have access, an absolute increase of 30% from 2000 to 2009 [1]. Given the widespread availability of internet access and the decreasing costs of video communication via computer, it is important to understand preferences and potential barriers, from the patient perspective, to facilitate telemedicine implementation.

Telemedicine can be employed in a synchronous or asynchronous manner [2]. In asynchronous telemedicine, also known as “store and forward,” health information is obtained and communicated between visits, at which point discussion of that information may occur. This aspect of telemedicine is already commonly practiced in many settings; examples include remote transmission of information from specialists (such as radiology) as well as patient use of email or weblogs for conveying information. By comparison, synchronous telemedicine involves real time interaction between provider and patient, such as by telephone or video. Use of this method is growing in several settings as follows: providing acute decision making, such as in telestroke [3]; facilitating intermittent, low-level management of chronic illnesses; increasing accessibility for patients who are either geographically isolated or find local travel challenging; enabling patients with uncommon diseases to access specialists [4, 5].

The potential benefits of synchronous telemedicine include improved access to healthcare, reduced waiting times for appointments, and increased patient adherence to chronic illness treatment plans. Cost savings associated with telemedicine have been estimated at over $4 billion [5]. Chronic diseases account for 75% of healthcare spending and represent a key target for both cost reduction and care improvement via telemedicine [6]. Although payer organizations have been reluctant to accept telemedicine due to uncertainties in its efficacy, an increasing body of literature demonstrates that telemedicine may be as effective (and in some cases superior to) the current standard of care in the treatment of chronic diseases such as diabetes, hypertension, and AIDS [4].

Obstructive sleep apnea (OSA) is present in 4–20% of adults (depending on the study and the operational definition) and remains underdiagnosed [7, 8]. OSA is often comorbid with other chronic conditions such as heart disease and hypertension, and requires long-term management [9]. Home sleep testing devices are increasingly common [10], and even telemetric continuous positive airway pressure (CPAP) titration has been shown to be effective [11]. The chronic management of OSA is a promising new application of telemedicine. Indeed, recent evidence supports the use of video visits in OSA management [12]. Patients receiving in-person visits or video visits were equivalent in terms of satisfaction and treatment adherence. We undertook the current study to answer three important questions regarding patient perceptions of in-person versus telemedicine forms of patient care in an academic Sleep Disorders clinic population as follows: (1) What are the barriers to in-person clinic visits? (2) What are the preferences regarding different telemedicine strategies? (3) What copay range do patients find acceptable for video-visits?

## 2. Methods

### 2.1. Survey

 We administered a brief survey, consisting of 14 multiple choice questions and one open-ended free text question, to patients seen in the Sleep Disorders Clinic at our institution between 2009–2011 (see Supplemtaery Material at doi:10.5402/2012/135329). The survey included questions pertaining to waiting times to be seen at primary care and specialist offices as well as communication practices, frequency, and preferences in regards to email, phone, video chat, and Patient Gateway (see Supplemtaery Material). Patient Gateway is a local service offered by Partners Health Care System (Massachusetts General Hospital and Brigham and Women's Hospital MA, USA) that allows patients to electronically communicate with their doctors through a secure platform. The survey was conducted between November 2011 and February 2012. We included adult Sleep Disorder patients who had been seen by one of two board-certified sleep neurologists at Massachusetts General Hospital in Boston, Massachusetts for a variety of complaints (the majority being for sleepiness, sleep apnea, and insomnia evaluations). This is an academic center serving a range of patient demographics; most reside in Massachusetts, with a minority traveling from neighboring states. We sent an email survey to patients if they had a viable email address on file (*n* = 282). A reminder email was sent one week later. An additional 156 patients, who did not provide an email address during routine clinical intake, were mailed a hardcopy of the questionnaire. These values do not include the 18 patients with invalid emails and the four patients for whom the hardcopy survey was returned due to invalid mailing address. Participation in the survey was voluntary, and no compensation was provided. The Partners Research Committee determined that this study was exempt from Institutional Review Board approval; patients provided implied consent by their participation in the survey.

### 2.2. Analysis

Study data were collected and managed using REDCap (Research Electronic Data Capture) software developed at Vanderbilt University, and hosted at Massachusetts General Hospital [13]. REDCap is a secure, web-based application designed to support data capture for research studies. REDCap provides an intuitive interface for validated data entry; audit trails for tracking data manipulation and export procedures; automated export procedures for seamless data downloads to common statistical packages; procedures for importing data from external sources. Survey responses were analyzed with Prism (GraphPad software, La Jolla, CA, USA). The Kruskall-Wallis nonparametric test (with Dunn's post hoc test) was used for group comparisons. For some survey questions, we combined certain answers in order to preserve the ordinal nature of the list for statistical analysis. For example, the question regarding comfort with video telemedicine, we combined the “not comfortable” with “not sure,” which is a conservative change regarding estimation of opinion regarding video.

To address the question of whether nonresponders differed in terms of age or sex, we selected a random sample of 50 nonresponders from the email cohort and the mail cohort. We found no significant difference in response rate according to sex for either mail or email surveys (*P* > 0.05, Fisher's Exact Test). For the email survey cohort, we found no difference in age between responders and the sample of nonresponders. However, for the mailed survey cohort, the respondents were older than nonresponders (62 versus 51 years of age; *P* < 0.05, ANOVA with Bonferroni correction).

## 3. Results

We received a total of 123 survey responses. The response rate from those who were emailed was similar to those who received the survey by postal mail (30% versus 24%, *P* > 0.2, Fisher's Exact Test), yielding an overall response rate of 28% (Table 1).

**Figure 1:**
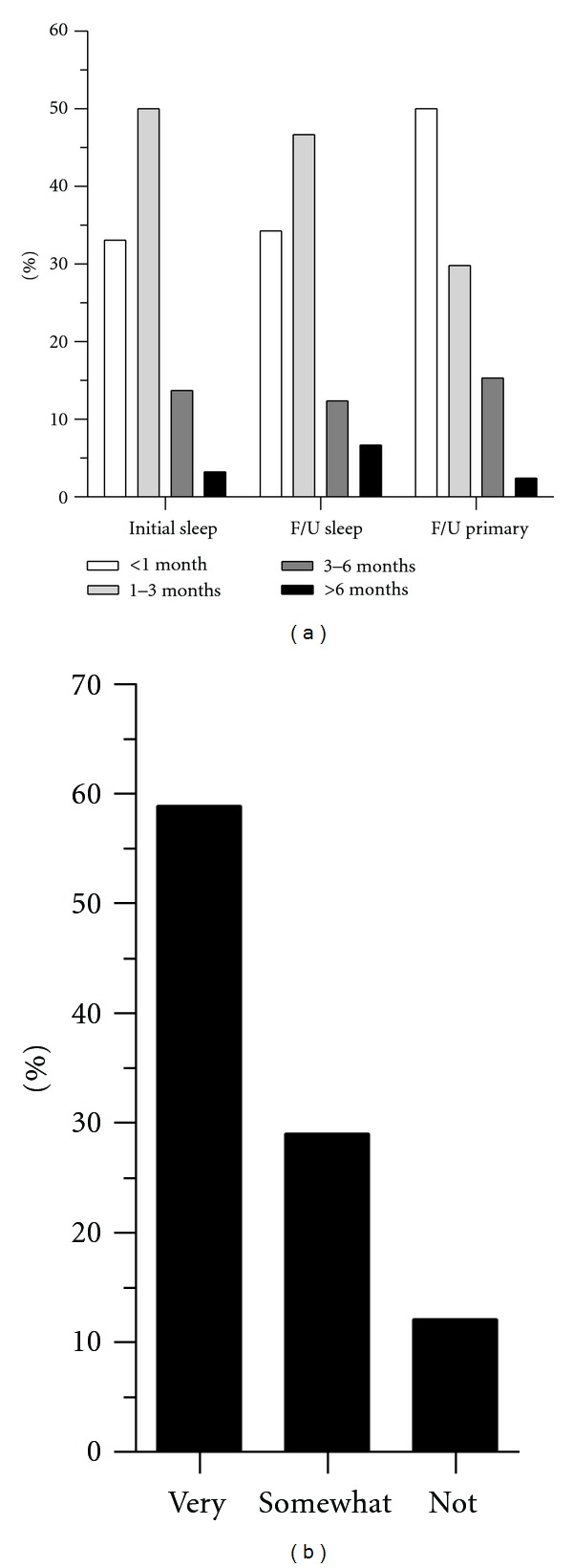
Waiting time for clinic visits (a) Waiting times for initial consultation and follow-up visits in sleep clinic (F/U Sleep) and follow-up visits in primary care (F/U Primary). For the follow-up visits, only the 84% of the population responding that had a follow-up visit (at the time of the survey) are included. (b) The percentage of respondents indicating their level of satisfaction with their waiting time to be seen in the Sleep Clinic.

83% of patients reported waiting under three months to be seen for their initial sleep consultation and for their follow-up visits (Figure 1(a)). The waiting time for follow-up appointments with primary care physicians was similar, except that waiting times of less than 1 month were more common. Satisfaction with Sleep Clinic waiting times was modest, with 41% of patients reporting they were either somewhat satisfied or not satisfied (Figure 1(b)).

 Patients indicated several important concerns regarding routine in-person clinic visits (Table 2). The most common challenges for face-to-face appointments were cost of parking (44%), time away from work/school (34%), cost of gas (26%), and requiring family or other support to travel (19%). About 28% of patients reported they were sometimes or frequently late for in-person appointments (data not shown).

**Figure 2:**
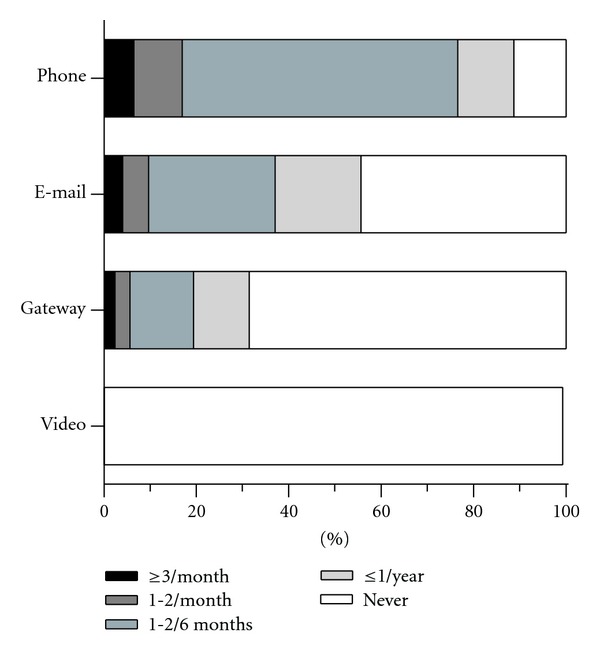
Frequency of use of different forms of telemedicine. Bar length indicates the percentage of respondents indicating communication with their provider(s) via telephone, e-mail, Patient Gateway (encrypted email platform; see Section 2), and video. The shading represents the frequency with which each method is utilized.

Telephone contact was the most common form of telemedicine, with a large majority (89%) of respondents employing this method at least 1-2 times per six months (Figure 2). More than half of respondents reported contacting their doctors by email, with the most common frequency being 1-2 times per six months. About 30% of respondents reported using the encrypted electronic e-mail communication platform (see methods). Only 20% of those surveyed reported using a health diary as part of their care plan (data not shown).

**Figure 3:**
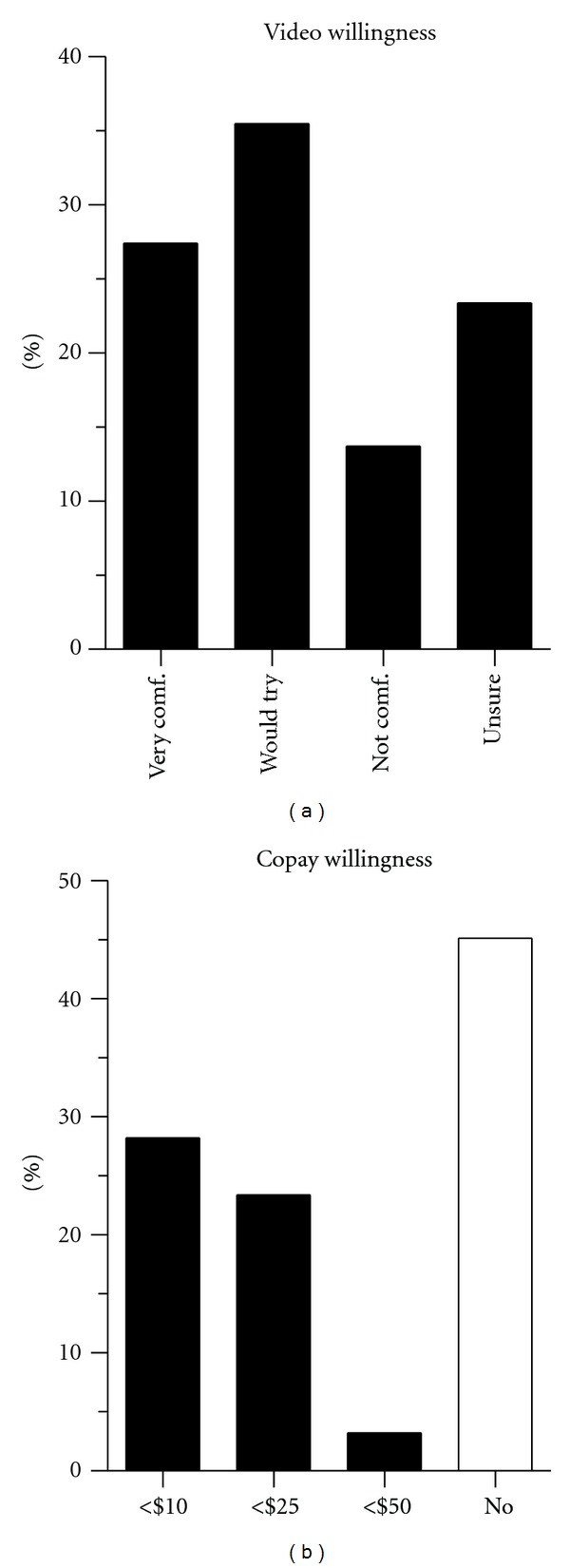
Patient willingness to utilize video visits (a) Percentage of patients reporting comfort levels with video-based follow-up appointments in the Sleep Clinic as follows: very comfortable (very comf.), would try, not comfortable (not comf.), or unsure. (b) Percentage of respondents who were willing to pay different copay amounts for video-chat appointments (US dollars).

Despite the lack of experience using video for clinical purposes, the majority of patients (63%) reported being very comfortable or willing to try this method of telemedicine (Figure 3(a)). More than half (54%) of respondents indicated they would be willing to pay a copay for a video appointment, and not surprisingly, they felt $10 or $25 was preferable to a $50 copay (Figure 3(b)). Of the 14% of respondents who were uncomfortable with video communication, the two most common reasons were that in-person visits feel more natural and that the doctor might need to perform an examination (Table 3). Only 13% of respondents felt video technology was too difficult or reported that they did not have a computer or internet connection.

 We tested whether respondent comfort with video telemedicine was related to age, sex, internet availability, willingness to pay a copay, or availability of an email address on file. For this analysis, we combined the “uncertain” and “not comfortable” responses (see Figure 3(a)), a conservative assumption that allowed the responses to remain ordered for statistical testing (nonparametric ANOVA). There was no difference in comfort level with video telemedicine by age, sex, or by method of survey response (mail versus email). Of those who reported being uncomfortable or unsure about video telemedicine, 11% reported lacking a computer or internet access as a reason, and only 2% indicated it was too difficult. This subgroup of responders was also less willing to pay a copay (*P* < 0.001).

## 4. Discussion

This survey study indicates that a substantial portion of patients seen in an academic Sleep Disorders clinic are willing to consider video telemedicine as an option for their care. Patients identified several practical barriers to in-person visits, including cost and inconvenience. Their main concerns regarding video visits, including feeling less natural and the need for physical examination, are already inherent in telephone communication, which they reported commonly utilizing. These results are encouraging for the development of video appointments in Sleep Medicine and is consistent with previous evidence that patients find tele-consultation equivalent to in-person consultation [14, 15], as well as a forward-looking editorial suggesting the importance of telemedicine in the care of sleep disorders [16].

### 4.1. Patient Perspective

Telemedicine provides both direct and indirect benefits to patients. Direct benefits include convenience, decreased waiting time, and increased specialist availability. Indirect benefits include avoidance of barriers to in-person visits, such as the time and cost associated with travel or missed work. To decrease wait times by changing the medium of communication from in-person to virtual, either the visit duration would have to be shorter or the filling of cancellations would have to be more efficient.

 Regarding the sense that video chat does not seem as natural as in-person visits, it is worth mentioning that phone and email, which are arguably even less natural-feeling, were commonly utilized by this cohort. It is possible that sentiments towards video communication will evolve to be even more accepted by patients as it becomes commonplace. Regarding the concern that the doctor might need to perform some physical examination, in sleep medicine it may be that certain stable patients could be adequately assessed without performing a physical exam that requires the physician to be present in the same room. In addition, certain elements of the examination such as weight or blood pressure could be performed at home or at a local health clinic. Additionally, many patients using video visits reported the absence of a physical exam to be acceptable and gave the experience high satisfaction scores [12]. Another area of concern involves confidentiality and privacy [17]. Although we did not assess this aspect directly, one respondent did express privacy concerns in the free text field. It remains unclear whether privacy is a substantial but unvoiced concern in this population.

The majority of patients were willing to pay either $10 or $25 per video appointment. With the increasing evidence of the treatment efficacy and patient support of telemedicine in a variety of medical settings, Sleep Medicine may experience similar benefits in terms of their chronic management. The extent to which third party payers might reimburse for virtual visits in Sleep Medicine remains uncertain but there are emerging mechanisms for supporting these types of visits financially if they can be shown to lower costs.

### 4.2. Physician Perspective

Physician acceptance of telemedicine incorporates personal preference, prior experience, reimbursement potential, and demonstration of improved or equivalent satisfaction and outcomes compared to in-person visits. Telephone contact with patients between clinic visits is employed across many specialties, especially for low-level decision making. The majority of physicians do not charge for telephone services, yet most are in agreement that compensation for their time is appropriate, whether in person or electronically [5]. Concerns about telephone-based care from the physician standpoint include limited access to the patient's health record, legal concerns about advice delivered in this setting, and challenges of documentation [18].

In specialties with particularly nuanced and complex examinations, such as neurology, telemedicine for diagnostic purposes has demonstrated good acceptance as well as inter-rater reliability [19]. Neurologists were also reported to universally feel that they were able to appropriately communicate management advice to their patients by video [14]. In Sleep Medicine, the extent to which physical examination influences chronic decision making in common disorders such as insomnia and sleep apnea remains untested.

### 4.3. Cost Effectiveness

In regards to the rising costs of health care, telemedicine may be a promising solution, as many studies have shown scheduling video chat appointments results in cost-savings for patients and hospitals. In one economic review of video telemedicine, 22 of 36 studies reported this method to be more cost effective than standard office appointments [5]. Video appointments could address a variety of the burdens associated with in-person visits listed by patients in this survey. For example, the time, cost, and inconvenience associated with travel could be ameliorated by a video visit option (Table 2), as has been reported for example in pediatrics [20] and oncology [21]. In addition, the decreased utilization of scarce outpatient practice space due to offloading to telemedicine-based practice could free up space for patients requiring in-person consultation. Telemedicine visits might also be useful for deciding which chronic patients require further in person care.

### 4.4. Opportunity in Sleep Medicine

Sleep medicine may be an ideal specialty in which video visits could accommodate routine chronic follow-up appointments. Patient-specific data, such as diaries for the insomnia patient and CPAP machine downloads for the OSA patient, could be reviewed at such visits, as could routine challenges with medications or equipment. Seeing the patient in their home environment may assist the clinician in more accurately diagnosing reasons for sleep dysfunction including ill-fitting equipment, certain aspects of poor sleep hygiene, or an overly illuminated bedroom. In a recent editorial, telemedicine was proposed as an important method to approach the ongoing challenges of cost-effective and outcomes-oriented chronic care of patients with sleep disorders. One study recently reported the successful use of video telemedicine in OSA patients, with similar treatment adherence and satisfaction levels compared to those with in-person visits [12]. This opportunity may extend to management of other chronic diseases in which evaluations and decision making may be considered using only remotely obtainable information.

## 5. Conclusion

The main limitations of this study include the 28% response rate and the single-center population. Thus the extent to which our findings generalize to other populations remains uncertain. Nevertheless, our findings highlight several important points and lay the groundwork for future development of Sleep Telemedicine. Patients experience multiple barriers to in-person visits that could be circumvented with virtual visits, and the majority of respondents were willing to pay a copay for a video visit despite none of the respondents having any personal experience with video telemedicine.

## Supplementary Material

The survey is consisting of 14 multiple choice questions and one open-ended free text question, to patients seen in the Sleep Disorders Clinic at our institution between 2009–2011.

## Figures and Tables

**Table 1:** Survey response rates.

Method	# Mailed
Sent via US mail	156
Replied via US mail	38
Response rate	24.4%

Sent via e-mail	282
Replied via e-mail	85
Response rate	30.1%

Total sent	438
Total responded	123
Response rate	28.1%

**Table 2:** Challenges with in-person visits.

Barrier	%
Cost of parking	43.9
Time away from work/school	34.1
Cost of gas	26.0
I require family or other support to travel	18.7
Time away from family	6.5
Cost of public transportation	5.7
Hard to find transportation	6.5

**Table 3:** Challenges regarding video telemedicine.

Reason	%
In-person visits feel more natural	47.6
My doctor may need to examine me (take my blood pressure, use a stethoscope, etc.)	23.8
I do not have a computer or internet	11.1
Video chat technology is too hard	1.6
Other^∗^	15.9

^∗^Examples: I do not have a web cam, do not have a laptop with video chat technology, privacy is an issue and if I am going to invest time in spending time with a Dr., then I might as well go to their office, technical difficulty with being online at same time (as the doctor).
