# Supplementary material for: Sleep Telemedicine: A Survey Study of Patient Preferences
Source: ISRN Neurol. 2012 Jul 9;2012:135329. doi: 10.5402/2012/135329 (PMC3400365; doi:10.5402/2012/135329)
Supplement: Supplementary file 1 — The survey is consisting of 14 multiple choice questions and one open-ended free text question, to patients seen in the Sleep Disorders Clinic at our institution between 2009–2011. [file 135329.f1.pdf]

Sleep Clinic Survey

Thank you for participating in this survey - you are helping us improve the care we deliver to our sleep patients!

How many months did you have to wait for your inital visit in the Sleep Clinic?

- ☐ Less than 1 month
- ☐ 1-3 months
- ☐ 3-6 months
- ☐ More than 6 months

How many months was the wait for your first follow-up visit at the Sleep Clinic?

- ☐ Less than 1 month
- ☐ 1-3 months
- ☐ 3-6 months
- ☐ More than 6 months
- ☐ I have not had a follow-up visit

How satisfied are you with the waiting time to be seen in the Sleep Clinic?

- ☐ Very satisfied
- ☐ Somewhat satisfied
- ☐ Not satisfied

How many months do you typically wait for a follow-up visit with your primary care doctor?

- ☐ Less than 1 month
- ☐ 1-3 months
- ☐ 3-6 months
- ☐ More than 6 months

How often do you contact a doctor's office by phone?

- ☐ Once per year or less
- ☐ 1-2 times per six months
- ☐ 1-2 times per month
- ☐ 3 or more times per month
- ☐ I do not contact my doctors by phone

How often do you contact a doctor by email?

- ☐ Once per year or less
- ☐ 1-2 times per six months
- ☐ 1-2 times per month
- ☐ 3 or more times per month
- ☐ I do not contact my doctors by email

How often do you contact a doctor by Patient Gateway email?

- ☐ Once per year or less
- ☐ 1-2 times per six months
- ☐ 1-2 times per month
- ☐ 3 or more times per month
- ☐ I do not use Patient Gateway

How often have you used a health diary on the advice of a doctor?

- ☐ Once per year or less
- ☐ 1-2 times per six months
- ☐ 1-2 times per month
- ☐ 3 or more times per month
- ☐ I have not used a health diary

How often do you contact a doctor by video-chat?

- ☐ Once per year or less
- ☐ 1-2 times per six months
- ☐ 1-2 times per month
- ☐ 3 or more times per month
- ☐ I do not contact my doctors with video chat.

How comfortable would you feel having a video-chat option for your Sleep Clinic follow-up appointments?

- ☐ Very comfortable
- ☐ I would try it
- ☐ Not comfortable
- ☐ Not sure

If you are not comfortable, what aspects of an in-person visit are most important that you feel would be missing? (Please check all that apply)

- ☐ In-person visits feel more natural
- ☐ My doctor may need to examine me (take my blood pressure, use a stethoscope, etc.)
- ☐ Video chat technology is too hard
- ☐ I don't have a computer or internet connection
- ☐ Other

Please explain. ______________________________

What problems do you face with traveling to MGH for doctor appointments? (Please check all that apply)

- ☐ Hard to find transportation
- ☐ Cost of gas
- ☐ Cost of parking
- ☐ Cost of public transportation
- ☐ Time away from work/school
- ☐ Time away from family
- ☐ I require family or other support to travel

How often have you been late or missed your visit to an MGH doctor because of transportation problems (such as traffic, parking, etc.) ?

- ☐ Frequently
- ☐ Sometimes
- ☐ Never

Would you be willing to pay a co-pay for a video chat appointment?

- ☐ Yes, if under $10
- ☐ Yes, if under $25
- ☐ Yes, if under $50
- ☐ No

Please provide us with any further comments you'd like to make.

____
